# Feature-by-Feature – Evaluating *De Novo* Sequence Assembly

**DOI:** 10.1371/journal.pone.0031002

**Published:** 2012-02-03

**Authors:** Francesco Vezzi, Giuseppe Narzisi, Bud Mishra

**Affiliations:** 1 Department of Mathematics and Informatics, University of Udine, Udine, Italy; 2 Institute of Applied Genomics IGA, Udine, Italy; 3 Courant Institute of Mathematical Sciences, New York University, New York, New York, United States of America; 4 NYU School of Medicine, New York University, New York, New York, United States of America; University of Chicago, United States of America

## Abstract

The whole-genome sequence assembly (WGSA) problem is among one of the most studied problems in computational biology. Despite the availability of a plethora of tools (*i.e.*, assemblers), all claiming to have solved the WGSA problem, little has been done to systematically compare their accuracy and power. Traditional methods rely on standard metrics and read simulation: while on the one hand, metrics like N50 and number of contigs focus only on size without proportionately emphasizing the information about the correctness of the assembly, comparisons performed on simulated dataset, on the other hand, can be highly biased by the non-realistic assumptions in the underlying read generator. Recently the Feature Response Curve (FRC) method was proposed to assess the overall assembly quality and correctness: FRC transparently captures the trade-offs between contigs' quality against their sizes. Nevertheless, the relationship among the different features and their relative importance remains unknown. In particular, FRC cannot account for the correlation among the different features. We analyzed the correlation among different features in order to better describe their relationships and their importance in gauging assembly quality and correctness. In particular, using multivariate techniques like principal and independent component analysis we were able to estimate the “excess-dimensionality” of the feature space. Moreover, principal component analysis allowed us to show how poorly the acclaimed N50 metric describes the assembly quality. Applying independent component analysis we identified a subset of features that better describe the assemblers performances. We demonstrated that by focusing on a reduced set of highly informative features we can use the FRC curve to better describe and compare the performances of different assemblers. Moreover, as a by-product of our analysis, we discovered how often evaluation based on simulated data, obtained with state of the art simulators, lead to not-so-realistic results.

## Introduction

*De novo* whole-genome sequence assembly (WGSA) is the task of reconstructing the genome sequence from a large number of short sequences (*i.e.*, *reads*) with no additional locational information or knowledge of the underlying genome's structure. Practically all the assemblers are based on a simple heuristic that if two reads share a sufficiently long subsequence (a prefix “matching” a suffix) then they can be assumed to have originated from the same locations in the genome. Thus, assembly would be a trivial task, if each read had unique placement on the genome and all the reads have been correctly read. This assumption is unfortunately foiled by various imperfections: all genomes contain exact or almost-exact repeats (with varying sizes and copy-numbers), all diploid genomes contain homologous autosomes making haplotype assignment ambiguous and all available sequencing technologies generate reads that are subject to sequencing errors of various kinds (*e.g.*, incorrect bases, homopolymer compressions, indels, *etc.*). When reads are available with high-coverage, pre-processing with repeat-maskers, 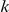-mer based error corrections or gap-filling attempts to overcome these hurdles using various heuristics, but only with limited success. Improved base-callers (using a Bayesian priors on the genome's base distributions) and novel assembly methods (combining short- and long-range information in a *dovetail* fashion) have been more effective in improving base-accuracy and in resolving repeat boundaries [1], [2].

For more than 20 years, Sanger sequencing has been the unquestionable method of choice in almost all the large genome projects. Many software pipelines (built around an *assembler*) have been proposed and implemented to tackle the hard task of assembling reads produced by such projects. The last few years have seen an unbridled and explosive innovations in the so-called Next Generation Sequencing (NGS) technologies. New sequencers (now often renamed Second Generation Sequencers in order to distinguish them from other newer technologies under development) possess the ability to sequence genomes at massively deep coverage at a fraction of the time and cost needed by Sanger Sequencing, thus opening up the opportunities for genome-wide association studies (GWAS), population genomics, characterization of rare polymorphisms, and personal clinical genomics. The main obstacles hindering such new technologies, however, are their limited ability to produce reads of length and quality comparable to the ones produced by Sanger-technology. Deluged by the incredibly high coverage data, but hampered by their poor quality, many new assemblers for short reads have resorted to filtering the reads into compressed graph structures (usually a de-Bruijn graph) and additional heuristics for error correction and read-culling (*e.g.*, dead-end elimination, p-bubble detection, *etc.*). Several de novo projects have been launched with some success in order to handle such short sequences as best as possible [3]. It is now commonly accepted that short reads make the assembly problem significantly harder [4], yielding final genome-assemblies of dubious value. To make matters worse, NGS data are often characterized by new and hitherto-unknown error structures, which can easily change within a year.

For a perspective on the difficulties facing genome-assembly projects, consider the draft sequence of the Human Genome [5], which was released in 2001, and took several large teams more than five years to finish and validate (but only at a genotypic level). Despite the time and financial resources engaged in such a processes, it must be stressed that it involved largely a manual process. In contrast, most of the current genome projects lack both time and money, forcing the developers to simply leave the assembly at a draft level (with many gaps and unresolved phasings).

Thus, it is timely to investigate independent means of assessing the assemblers' capability for correct de novo genome assembly. Most of the traditional metrics used to evaluate assemblies (N50, mean contig size, *etc.*) emphasize only size, while nothing (or almost nothing) is said about how correct the assemblies are. A typical such metric (especially, in the NGS context) consists in aligning contigs back to an available reference. However, this naïve technique simply counts the number of mis-assemblies without attempting to distinguish or categorize them any further. Moreover, such tests are usually performed on simulated datasets [6] leaving open the question of how realistic these datasets are.

In Phillippy et al. [7], a tool dubbed *amosvalidate* was proposed with the aim of identifying a set of features that are likely to highlight assembly errors. The *amosvalidate* pipeline returns for each contig its “features” – contigs or contigs' fragments containing several different features suggest their “mis-assemblies” (*i.e.*, errors). The *amosvalidate* pipeline has been recently used by [8] in order to compute the Feature Response Curve (FRC) [8]. This metric characterizes the sensitivity (coverage) of the contigs as a function of its discrimination threshold (number of features). FRC can be used to compare different assemblers, emphasizing both contig-sizes as well as correctness. It also has the advantage over other common metrics for comparing assemblies that no reference sequence is required at all.

A still unexplored area is the relationship (*e.g.*, statistical independence) among the different features. Note that FRC simply counts the features in each contig but it cannot account for the correlations among the different features, thus biasing the results in favor of assemblers that emphasize certain over-represented dimensions of the feature-space. For example, a contig with two highly correlated features should be weighted differently from two contigs each containing only one type of features. Moreover, the *amosvalidate* pipeline computes 11 different features, whose likely redundancy makes their interpretation biased and counter-intuitive. Furthermore, as the structure of the feature-space is better understood, it is hoped that well-chosen feature-combinations would suggest good “score” functions that can be globally optimized (*e.g.*, by algorithms like SUTTA [1]) to improve assembly accuracy (since they would minimize features in the resulting assemblies). In this way, sequence-assembly problem can then be formulated as a (supervised) machine-learning problem, whose goal is to select the best subset of features distinguishing better assemblies from marginal ones.

The aim of this paper is thus twofold: Analyze the relationship among the different features in order to understand how they are correlated. Next, select a small number of (non-redundant) features capable of characterizing the correctness of an assembly. For the first task we will use Principal Component Analysis (PCA) to *extract* new *synthetic features* while, for the second task we will use Independent Component Analysis (ICA) to *select* a small set of informative features. As a consequence of our analysis, we can highlight the differences between the synthetic features obtained from real data sets versus the ones obtained from simulated datasets, and thus, gauge reliability of empirical analyses based on simulated data.

### Assembly Features

More than 20 assemblers have been designed specifically for short reads in the last two years – a large fraction of them for just Illumina platform. In a relatively short period, these new pipelines more than doubled the population of assemblers, which were built primarily to handle data from Sanger based projects. As noticed by the authors, Miller et al. [9], each of these new assemblers appeared to be claiming better performance relative to the others that preceded it. However more-often-than-not the only feature to have been judged was contig size or features closely related to it. When a reference sequence was available, contigs aligned against the reference gauged mis-assemblies; often, these empirical analysis were built on simulated “in vitro reads.” Despite many such attempts to quantify the quality of the assembly, there is no evidence of their universal applicability – for instance, will it be reasonable to expect the same behavior from an assembler, when run on an utterly different organism or on some other real data? Moreover, the empirical results from such simulated experiments seemed to vary with the read simulator being used and its capabilities to effectively reproduce in-vitro datasets' verisimilitude to the real genomes and sequencers.

In Lin et al. [6] 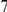 NGS assembler have been evaluated by assembling 8 different organisms ranging from 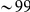 kbp (base pair) to 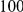 Mbp using varying coverages (from 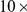 to 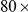) and read lengths (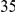 and 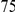 bp). Authors evaluated standard metrics like N50, sequence coverage (the percentage of reference genome covered by assembled contigs) and rates of mis-assembly. However, since results of this kind are deeply connected to the statistics of simulated reads, suitably chosen (most likely, non-realistic) simulation could produce as optimistic (or pessimistic) results as desired.

More recently two independent groups have started competitions aimed to comprehensively assess state-of-the-art *de novo* assembly methods when applied to current next-generation sequencing technologies. Specifically, the Assemblathon 1 [10] has been able to highlight that it is now possible to assemble a relatively large genome with a high level of coverage and accuracy, but, as previously shown also in [8], large differences exist between the assemblies. Moreover such evaluation was performed only on one simulated data set which clearly limits the extension to which the conclusions can be generalized. Similarly, the GAGE evaluation team [11], evaluated several leading *de novo* assembly tools on four different data sets of Illumina short reads. Their conclusions are that data quality, more than the assembler itself, is important to achieve high assembly quality, and also that there is an high degree of variation in the contiguity and correctness obtained by different assemblers.

The metric used to judge assemblies is too frequently just the contig size: usually longer contigs are preferred to shorter ones (statistics like N50 stress the presence of long contigs, as it thresholds the contigs above a value, called N50, such that the selected sets add up to 50% of the total genome size). In Phillippy et al. [7], the authors propose a more intelligent approach to better inform the overall assembly quality and correctness: *de novo* assembly is based on the double-barreled shotgun process, therefore the layout of the reads, and implicitly, the layout of the original DNA fragments, must be consistent with the characteristics of the shotgun sequencing process. In particular the authors noticed that sequences of overlapping reads must agree and that the distance and the orientation between mated reads must correspond to the expected statistics. They noticed that mis-assembly events fall into two major categories: repeat collapse/expansion and sequence rearrangement. In the former case, the assembler fails to correctly estimate the number of repeats in the genome, while, in the latter case, the assembler shuffles (translocates or inverts) the order of multiply repeated copies. So far, these features have been based on assembly of a genotypic sequence, though their extensions to haplotypic sequences can be achieved *mutatis mutandis*.

Single Nucleotide Polymorphisms (SNPs) are usually good indicators of collapsed or mis-assembled regions. In fact, since single base read errors occur uniformly randomly (i.i.d.), while SNPs can be identified by their correlated location across multiple reads, a collapse (or an expansion) can be recognized by the local variations in coverage. A missing repeat causes reads to stack up in the remaining copies, increasing the read density locally. Conversely, a repeat expansion causes a reduced read density among the copies.

Mate pairs highlight incorrect rearrangements: these events are identified by the associated pair of reads being too close to or too distant from each other, mate pairs orienting in wrong directions or reads with an absent mate (in the assembly) or a mate in a different (wrong) contig. Obviously, multiple mate-pair violations are expected to co-occur at a specific location in the assembly in the presence of an error.

Another important way to asses assembly correctness, as introduced in Phillippy et al. [7], relies on 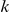-mers (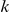-length words). By comparing the frequencies of 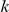-mers computed within the set of reads (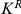) with those computed solely on the basis of the consensus sequence (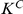), it is also possible to identify regions in the assembly that manifest an unexpected multiplicity. For each 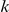-mer in the consensus, the ratio 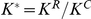 is computed. 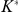 has an expected value close to the average depth coverage. Positions in the consensus where 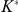 differs from expected values can be hypothesized to have been mis-assembled. Further information can be extracted from unassembled reads (*i.e.*, leftovers). Unassembled reads that disagree with the assembly can reveal potential mis-assemblies.

Summarizing, the tool (*amosvalidate*), proposed by Phillippy [7], computes a set of features (listed below) in order to asses the overall assembly quality and correctness. In particular *amosvalidate* pipeline identifies the following 12 features:

1. BREAKPOINT: Points in the assembly where leftover reads partially align;
2. COMPRESSION: Area representing a possible repeat collapse;
3. STRETCH: Area representing a possible repeat expansion;
4. LOW_GOOD_CVG: Area composed of paired reads at the right distance and with the right orientation but at low coverage;
5. HIGH_NORMAL_CVG: Area composed of normal oriented reads but at high coverage;
6. HIGH_LINKING_CVG: Area composed of reads with associated mates in another scaffold;
7. HIGH_SPANNING_CVG: Area composed of reads with associated mates in another contig;
8. HIGH_OUTIE_CVG: Area composed of incorrectly oriented mates (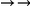, 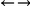);
9. HIGH_SINGLEMATE_CVG: Area composed of single reads (mate not present anywhere);
10. HIGH_READ_COVERAGE: Region in assembly with unexpectedly high local read coverage;
11. HIGH_SNP: SNP with high coverage;
12. KMER_COV: Problematic 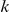-mer distribution.

It is suggestive of feature analysis that if a contig is found to contain several features (of different types), then a likely explanation could be found in the contig's mis-assemblies. Despite the indirectness of how features diagnose problems in assemblies, it represents a significant improvement over the simple but non-informative measures like N50 and mean contig length. However, the results from feature analysis are strongly dependent on how the features are combined, especially when the relationships among the features are ignored. It is expected that different features are symptomatic of different ways assemblers and their heuristics introduce different kinds of errors. Yet, it is not immediately clear how the simple feature counting can be used to compare the performances of two or more assemblers. Moreover, many features are intricately correlated, thus amplifying certain errors while subduing others into less prominence. For example, an area with high 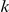-mer coverage is likely to contain many paired read features. This example raises the question whether it would be possible to concentrate the analysis to only a handful of meaningful features or use a linear combination of few such features to create newer and better set of synthetic and meaningful features.

In Narzisi and Mishra [8], a new solution was proposed to compare the assembly correctness and quality among several assemblers. After running *amosvalidate*, each contig is assigned the number of features that correspond to doubtful sequences in the assembly. For a fixed feature threshold 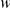, the contigs are sorted by size and, starting from the longest, only those contigs are tallied, if their sum of features is 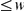. For this set of contigs, the corresponding approximate genome coverage is computed, leading to a single point of the Feature-Response curve (FRC). FRC allows to easily compare different assemblies by simply plotting their respective curves.

FRC can be applied to all the features or to a subset of them (or even just a single one, if a particular kind of error is of interest). It would be desirable to plot the FRC on a minimal subset of the most important features or on a small number of synthetic features capable of capturing the most important information (*i.e.*, variation). Moreover a rigorous study of the correlation among different features could give us information about the behaviours of available assemblers, and help us in designing new tools (*e.g.*, assemblers that maximize a score function based on the most predictive features).

For this purpose we used an unsupervised learning method in order to *extract* and *select* a subset of relevant features to understand their inter-relationships. We obtained several de-novo assemblies by assembling different genomes with a wide range of assemblers. For each assembly we extracted the 11 *amosvalidate*-features (HIGH_LINKING_CVG and HIGH_SPANNING_CVG have been collapsed into a single feature) and we used PCA and ICA (principal- and independent component analyses, respectively) to extract and select a set of synthetic features and a set of highly informative features, respectively (see Materials and Methods). Moreover we explored the relationship among the 11 *amosvalidate*-features and two additional commonly used metrics: N50 and number of contigs output (NUM_CONTIG).

When counting the number of features on a contig we used the following approach: single point features (SNP or BREACKPOINT) are counted as a single feature, while features that affect a contig's subsequences (*i.e.*, KMER_COV) of length 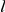 account for features, with 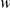 assuming a predefined threshold. In all our experiments 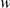 was kept fixed at 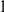 Kbp.

We also studied the relationships among features in two cases: long (*i.e.*, Sanger-like) reads as well as short (*i.e.*, Illumina-like) reads. Moreover, in each case we worked with both real and simulated datasets in order to quantify the differences between the features obtained from the two kinds of data.

## Materials and Methods

One of the main problems of data mining and pattern recognition is model selection that aims to avoid overfitting through model parsimony, which often involves dimensionality (or degrees-of-freedom) reduction. The key idea is to reduce the dimensionality of the dataset by sub-selecting only those features, which jointly describe the most important aspects of the data. Furthermore, dimensionality reduction allows a better understanding of the problem by focusing on the important *components*, and in highlighting hidden relationships among the variables. Recently, research focusing on dimensionality reduction has seen a renewed interest as their importance in both supervised and and unsupervised learning has become obvious. Techniques based on PCA, ICA, shrinkage, Bayesian variable selection, large margin classifiers, 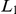 metrics, regularization, maximum-entropy, minimum description length, Kolmogorv complexity, *etc.* are all examples of Occam's razor, trimming away unnecessary complexity.

In the context of sequence metrics, our interests lie primarily in unsupervised learning approaches. Two main techniques can be used to reduce the dimensionality of a problem: feature extraction and feature selection. Feature extraction techniques combine available features into a new reduced set of *synthetic* features, representing the most important information. Among the most used techniques involving linear models, the following three dominate: Principal Component Analysis (PCA) [12], Independent Component Analysis (ICA) [13], and Multilinear Subspace Learning (MSL) [14].

Feature selection techniques focus on finding a minimal subset of relevant features containing the most important information in the dataset. Usually these methods try to select a subset of features that maximizes correlation or mutual information. Since this problem in general can be intractable, practical approaches are based on greedy methods that iteratively evaluate and increment a candidate subset of features [15]. Other common methods are based on Sparse Support Vector Machines (SSVM) [16], and PCA and ICA techniques, as discussed earlier [17], [18].

We chose to perform PCA in order to extract the most important components capable of succinctly describing assembly correctness and quality. PCA components emphasize the connections among features and their correlations. Moreover, the PCA results can be used to understand redundancy in a given set of features. Once PCA has established a high degree of redundancy, we can use ICA to select the most important features and then parsimoniously build on only those to compare assembly performances.

### PCA: Principal Component Analysis

Principal Component Analysis (PCA) is a popular multivariate statistical technique with many applications to a large number of disparate scientific disciplines [12]. It finds a set of new variables (Principal Components) that account for most of the variance in the observed variables. A principal component is a linear combination of optimally weighted observed variables.

PCA analyzes a matrix (*i.e.*, a table), whose rows correspond to observations and the columns, to the variables that describe the observations. In our case, the observations are *de novo* assemblies of different genomes performed with several assemblers, while the columns are the features describing the quality and correctness of the assemblies. PCA extracts the important information from the data table, and compresses the size of the table by keeping only the important information, thus simplifying the description of the data set. New variables, each one a linear combination of the original variables and called principal components (PCs), are computed in order to achieve these desiderata. The first PC is required to achieve the largest variance reduction (*i.e.*, the component that “describes” the largest part of the variance of the data set). The second component is computed under the constraint of being orthogonal to the first component, while accounting for the largest portion of the remaining variance. The subsequent components are computed with similar criteria.

PCs are described by eigen-vectors that represent the linear combination over all the original variables (*i.e.*, features). Eigen-vectors are ordered according to a monotonically decreasing order of eigen-values. The eigen-vector with the largest eigen-value explains the main source of variance, with the remaining ones explaining successively smaller sources of variance. PCA on a dataset described by 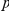 variables returns 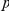 eigen-vectors (*i.e.*, 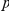 PCs). However, we are interested in keeping only those PCs that capture as much of the important information in the data as possible. A widely used rule of the thumb is to fix a variance threshold, which determines the eigen-vectors that can be safely discarded (*i.e.*, retain only those PCs that account for a certain amount of variance). A practically used heuristic value for variance threshold is often taken to be 80%. A more robust method is based on random matrix theory (RMT). By fitting the Marčenko-Pastur distribution [19] to the empirical density distribution of the eigen-values, one can determine the less informative eigen-vectors and discard them.

### ICA: Independent Component Analysis

Independent Component Analysis (ICA) is a signal processing technique that was originally devised to solve the *blind source separation problem*. ICA represents features as a linear combination of Independent Components [20]. Independent Components (ICs) have been used to select the most independent (*i.e.*, the most important) features [21].

ICA differs from other methods as it looks for components that are both statistically independent, and yet, non-Gaussian (*e.g.*, has non-vanishing high order moments – beyond mean and variance – such as the fourth-order moment, represented by kurtosis). Given a set of observations as a vector of random variables 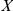, ICA estimates the Independent Components 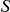 by solving the equation 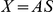 with 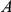 being the so-called *mixing matrix*. ICs represent linear combinations of features expressing maximal independence in the data. We followed the method described in [22] to select the most informative ICs by picking those with highest kurtosis (*i.e.*, the 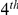 order cumulant). The underlying intuition is that higher is the kurtosis of an IC more “peaked” is its distribution, making it deviate further than what could be expected from central limit theorem (CLT). After selecting the ICs with kurtosis values in the top 80% of the kurtosis distribution, we singled out from each IC that feature, which contributed the highest in the linear combination.

### Experiments and Tools

We worked both with real and simulated datasets. We concentrated our attention on small bacterial and viral genomes for several reasons: first, the sample of assembled bacterial genomes is sizable enough to satisfy our aims; second, bacterial genomes are not diploid; and last but not least, the in silico experiments can be conducted with an affordable amount of resources (primarily, the computation time). For a detailed description of data used refer to supplementary file Document S1.

#### Long Reads

We downloaded from NCBI public ftp, data from 21 completed sequencing projects, consisting of reads, quality and ancillary data (paired read information, vector trimming, *etc.*). The organisms' genome lengths varied from 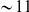 Kbp (*West Nile virus*) to 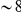 Mbp (*Bradyrhizobium sp. btai1*). All the 21 data sets have been assembled using 5 different de novo assemblers for long reads: CABOG [23], MINIMUS [24], PCAP [25], SUTTA [1], TIGR [26] for a total of 105 assemblies. Only 84 (CABOG 20, MINIMUS 15, PCAP 20, SUTTA 15, TIGR 14) were used in the subsequent analysis. We discarded 21 assemblies for two reasons: the assembler returned with error (missing data) and the assembly was clearly of bad quality (data outlier). Confronted with the first situation, we tried to resolve the problem by further manual interventions, but more often than not, we failed to understand the source of error, while in few other cases, usually the problem was due to bad format conversions (*e.g.*, CABOG was the only assembler unable to parse the ancillary file provided as input for *Staphylococcus aureus* dataset). In the second situation, we noticed that on some datasets (for example, *Bradyrhizobium sp. btai1*) some assemblers produced much worse results than the others (TIGR produced 19680 contigs while CABOG 72). Since both PCA and ICA were adversely affected by the presence of such outliers, which we assumed to be due to a wrong format conversion step, we disregarded these data points. All the assemblers have been tested using the default parameters, as provided by their implementers.

Another 20 bacterial organisms were selected to generate 20 simulated coverages. We used MetaSim [27] to generate a 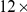 coverage composed of paired reads of mean size 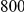 bp with insert sizes of length 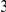 Kbp and 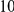 Kbp (forming respectively a 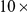 and a 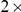 coverage) for each genome. These 20 sets have been assembled using CABOG, MINIMUS and SUTTA with default parameters, while PCAP has been used after relaxing some parameters (“-d 1000 −l 50 -s 2000”) in order to obtain results comparable to the other three assemblers. We did not use TIGR assembler in order to avoid its poor assembly results, which could not be corrected even after changing various parameters. Of the 80 assemblies produced, 4 failed. The 76 remaining assemblies did not create outliers (This can be seen as the first significant difference between analyses involving real and simulated datasets).

For each assembly we used all the 11 *amosvalidate* features and inserted them in a row in the experiment table, which has a row for each observation (*i.e.*, assembly) and a column for each feature. For the PCA analysis we also added two more columns: N50 and number of contigs (NUM_CONTIG).

#### Short Reads

We also performed a similar set of experiments for short reads. De novo assemblers for short reads have appeared only very recently and apart from the *multifasta* file containing all the computed contigs, no standard output format is provided. A particularly useful format used by all the Sanger based assemblers (mandatory for *amosvalidate*) is the afg format. An afg file is a text-based file that contains all the information related to reads, paired reads and contigs (in particular, the layout information, *i.e.*, where a read has been used in generating a consensus). This file is fundamental for running *amosvalidate* and hence, for the assembly features.

Velvet [28], SUTTA [1] and, RAY [29] natively create such files. In order to produce such files with other popular assemblers like ABySS [30] and SOAP [31], we found no solutions apart from mapping the reads back to the contigs and then use a program provided by the ABySS suite (*abyss2afg*) to obtain the afg file. Obviously, the layout created this way is unlikely to coincide with the real layouts. In particular, reads that fall in repeated regions are likely to mis-located, thus producing a “wrong” layout. However, since this was the only available method to obtain the layout files for ABySS and SOAP, we used these layouts for our analysis. We take this opportunity to strongly encourage that the afg files be provided by all the assemblers, in order to make them amenable to more accurate FRC analysis in the future.

Another stumbling block, we faced with short reads dataset, involved a lack of a sufficiently large corpora of genomes that have been assembled, *i.e.*, a paucity of a repository of short reads datasets for genomes. Data loaded on the Short Read Archive is obtained through different pipelines and different protocols, making it really hard to obtain several assemblies with several assemblers. A similar problem concerns the read length. Over the last two years Illumina reads have grown in length from 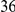 bp to around 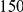 bp (or more), but often assemblers are optimized only for certain ranges of read lengths. Moreover, almost always raw reads were needed to be trimmed and/or filtered to remove contamination, which has invariably improved the final results.

Despite these practical difficulties, we assembled four real datasets: *Escherichia coli* (SRX000429) composed of paired reads of length 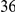 bp and insert size of 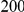 bp, *Chlamydia trachomatis* (ERX012723) composed of paired reads of length 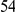 bp and insert size of length 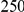 bp, *Staphylococcus aureus ST239* (ERX012594) composed of paired reads of length 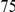 bp and insert size of 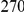 bp and, *Yersinia pestis KIM D27* (SRX048908) composed of reads of length 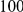 bp and insert size of length 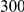 bp. In order to achieve a number of experiments that allowed PCA and ICA to yield statistically significant results, we assembled for each genome different random coverages ranging between 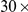 and 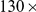. In order to assess parameters, for each genome, for each coverage and for each assembler, we varied the most important parameters and retained the results with the best trade-offs between N50 and number of contigs. We performed 105 assemblies and kept 82 of them (20 ABySS, 17 RAY, 20 SOAP, 9 SUTTA and, 16 VELVET) after discarding the outliers.

The same 20 genomes used to obtain the simulated datasets for Sanger were used also for Illumina. For each of the 20 genomes, we used SimSeq, the read generator used for Assemblathon 1 [10] (www.assemblathon.org), to produce an 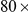 coverage formed by paired reads of length 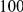 bp and insert size of 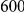 bp. For those experiments we used ABYSS, RAY, SOAP, and, VELVET. The most important parameter to set in those assemblers is the *k-mer* size, *i.e.*, the size of the word used to compute overlaps. We noticed that that by fixing this parameter to 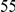 bp all the assemblers were able to achieve good and comparable results. We did not use SUTTA because the publicly available version was mainly designed for ultra-short reads (*i.e.* reads of length 36–55).

We produced two tables, one for the real data and one for the simulated data. For each assembly we computed 10 *amosvalidate*-features (BREAKPOINT feature could not be computed, since at present only SUTTA and VELVET return the unused reads). In the PCA analysis, we also added to those features the N50 and the number of contigs output.

## Results and Discussion

As explained in Materials and Methods, we used PCA in order to extract the most important Principal Components (PCs) and analyzed whether and how the features are correlated. In order to choose how many PCs to keep, we used random matrix theory (RMT) as suggested in [19]. In the following, we describe the results achieved with PCA on the different datasets. Moreover, we present the results achieved with ICA, in particular we show how the FRC can be used on a small subset of features to better describe the behavior of the different assemblers. To evaluate the results from this analysis, we used the reference genome in order to compute the number of *real* mis-assemblies by aligning the de novo contigs. We used *dnadiff* [7] in order to compute the mis-assembly. When parsing *dnadiff* results, we ignored small differences like SNPs and short indels, and disregarded breakpoints occurring within the first 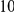 bp of a contig. This kind of analysis enabled us to gauge how well FRC represents the relationship between different assemblies/assemblers. In particular we could evaluate whether restricting the analysis to the ICA-feature space could improve the predictability of the assembly quality.

PCA was performed on the extended features space (*amosvalidate*-features plus N50 and NUM_CONTIG) while we restricted the analysis only to the *amosvalidate*-features for ICA. We focused specifically on the relation between the last two metrics, commonly used in judging assemblies, and their relation to the other features as well as the excess-dimensionality of the feature space.

Lest it appears strange that half of the results presented in this paper concerns the older Sanger Sequencing Technology, some remarks are in order. Although Sanger sequencing has been replaced by NGS approaches, we consider this empirical study to be of critical importance for the following statistical analysis, especially, if the features are to have a universal interpretation. Sanger sequencing is a well-known and stable method, used for more than 20 years, and the tools used to cope with Sanger data have been tested in a wide variety of situations. Thus, long reads present a useful benchmark in order to assess results. The utility of the long-read analysis is likely to become even more relevant in the near future, as all available NGS technologies have been improving in their read lengths, steadily approaching Sanger reads in length.

### Long Reads Results

**Figure 1:**
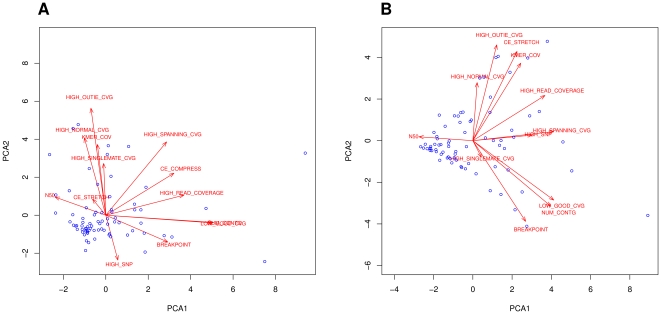
First PC versus Second PC: Long Reads Datasets. The plots in Figure A and B show the results of plotting the first principal component against the second computed on real and simulated datasets respectively. The blue dots represent the assemblies.

We performed PCA on the real as well as on the simulated dataset. In Figure 1, we plotted the first component versus the second in order to have a graphical representation of how the assemblies are separated by the first two PCs.

**Figure 2:**
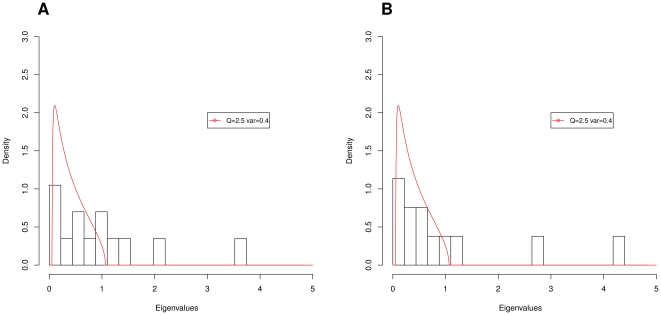
Marčenko-Pastur Distribution: Long Reads Datasets. We found the Marčenko-Pastur that best fits the eigen-value distribution. All eigen-vectors with eigen-values under the Marčenko-Pastur function are considered non informative. Figure A shows the results obtained on real long read, conversely Figure B shows the results obtained on simulated long reads.

In the real dataset, 6 PCs are necessary to represent at least 80% of the total variance, while in the simulated dataset only 5 PCs are necessary to represent the same amount of variance. A more careful analysis performed by fitting the Marčenko-Pastur distribution to the empirical density distribution of the eigen-values (see Figure 2), showed how to prune the eingen-vectors with eigen-values lower than one. This more precise analysis tells us that we need five and four PCs to fully describe the real and the simulated datasets, respectively. Both these methods also suggest how the feature space (11 *amosvalidate*-features plus N50 and NUM_CONTIG) is “over-dimensioned” and what can be eliminated without loss of valuable information. Examining the first eigen-vector (*i.e.*, first PC) of the real dataset closely, we see that the most important features are LOW_GOOD_CVG and NUM_CONTIG. The other positive contributing features are connected to the presence of areas with no uniform coverage. Surprisingly the acclaimed N50 metric not only lacked a large coefficient, but instead exhibited negative correlations with the others. This result suggests that high N50 values are simply a consequence of mis-assemblies and due to the fact that many assemblers try aggressively to merge as many sequences/sub-contigs as possible. In the second component, the main source of variation among assemblies with a large number of features is due to mis-assembled repeats (HIGH_READ_COVERAGE, K_MER_COV, HIGH_OUTIE_CVG, and HIGH_SPANNING_CVG), a low number of contigs and SNPs. The first three components account for the 55% of the total variation.

Examining the results from the simulated datasets, we noticed that no COMPRESSION feature has been found in any of the assemblies. The first eigen vector of the simulated dataset is similar to the ones obtained from real data, indicating a consistency between the two analyses. Again LOW_GOOD_CVG and NUM_CONTIG are among the most important features and N50 is again negatively correlated. The second component is similar to the one obtained from real data, as the main source of variation is again between assemblies characterized by repeats assembled in the wrong copy number and assemblies with too few contigs and breakpoints. The first three components account for 70% of the total variance.

A closer examination reveals that real and simulated PCs are somewhat different. Despite a complete absence of a feature in the simulated dataset (probably a failure of the read simulator to properly simulate the insert variation), we notice several differences: the first “simulated PC” gives non-negligible importance to features like STRETCH, HIGH_SNP, and KMER_COV that have much smaller importance in the first “real PC.” A similar situation holds true also for the second PC. The third components are utterly different (see Table 1), but not unexpected. We are thus led to conclude that sequence assembly evaluation based on simulated experiments could be misleading, unless genome sequence simulators are further improved.

**Table 1:** More Informative Principal Components For Long Reads.

	Real			Simulated		
FEATURES	PC1	PC2	PC3	PC1	PC2	PC3
BREAKPOINT	0.29	−0.14	−0.21	0.26	−0.38	−0.04
COMPRESSION	0.32	0.22	0.35	-	-	-
STRETCH	−0.06	0.08	0.27	0.22	0.42	0.12
HIGH_NORMAL_CVG	−0.10	0.40	0.21	0.02	0.2	−0.44
HIGH_OUTIE_CVG	−0.07	0.56	−0.09	0.12	0.46	0.01
HIGH_READ_COVERAGE	0.36	0.10	−0.13	0.36	0.21	−0.19
HIGH_SINGLEMATE_CVG	−0.01	0.27	−0.53	0.04	−0.07	−0.76
HIGH_SNP	0.05	−0.23	−0.13	0.30	0.02	−0.18
HIGH_SPANNING_CVG	0.28	0.38	0.31	0.41	0.04	0.00
KMER_COV	−0.03	0.37	−0.48	0.24	0.37	0.16
LOW_GOOD_CVG	0.50	−0.04	−0.02	0.41	−0.28	0.04
N50	−0.23	0.09	0.20	−0.27	0.01	−0.30
NUM_CONTG	0.50	−0.03	−0.02	0.39	−0.31	0.02
cumulative variation	27%	44%	55%	36%	59%	70%

First three PCs for the two long reads datasets: real long reads, simulated long reads. At the bottom of each component we reported the cumulative variation represented.

The principal component analysis (PCA) convinced us that the feature-space is highly over-dimensioned. Therefore we tried to *select* from the feature space the more informative features in order to estimate the performance of different assemblers on a small feature subspace. This analysis, leading to feature selection, was accomplished using another multivariate technique known as Independent Component Analysis (ICA). Following the method proposed in [22], we performed ICA using the *fastICA* algorithm on the *amosvalidate* features. We extracted the Independent Components (ICs) and selected the most representative feature in each of the ICs with the highest kurtosis value. From the real dataset, we selected the following 6 features: COMPRESSION, HIGH_OUTIE_CVG, HIGH_SINGLEMATE_CVG, HIGH_READ_COVERAGE, KMER_COV, and LOW_GOOD_CVG.

**Figure 3:**
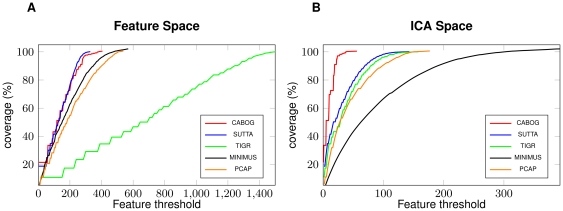
Feature Response Curve and ICA features: Long Reads. Figure A shows the FRC for the 5 assemblers on *Brucella suis* dataset when using all the feature space. Figure B shows the FRC computed on the ICA-selected features.

In Figure 3, we illustrate how the ICA-subspace allows better evaluation of different assemblers. Figure 3 shows the FRC for the assembly of the *Brucella suis* dataset computed on all the feature space (Fig. 3A) and on the ICA-feature space (Fig. 3B). In the first case, we see how, rather surprisingly, TIGR now behaves much worse than all other assemblers, while PCAP, MINIMUS, CABOG and SUTTA have comparable performances. It is surprising that TIGR performs worse than MINIMUS, which does not use the important information, available in paired reads. Inspecting these two assemblies closely (Table 2), we see how MINIMUS produces a highly fragmented assembly (206 contigs) in comparison to TIGR (69 contigs). If we plot the FRC after reducing the space to the only ICA-features (Fig. 3B) we obtain a slightly different picture. Looking only at the most informative features we discovered that CABOG performs better than all the other assemblers, while SUTTA, TIGR and PCAP are more or less equivalent. This picture is concordant with the results showed in Table 2, from which we clearly see that MINIMUS is the assembler with the poorest performance.

**Table 2:** Assembly Comparison Real Long Reads: Brucella suis.

Assembler	# Ctg	N50 (Kbp)	Max (Kbp)	Errs	# Feat	# corr Feat	# ICA Feat	# corr ICA Feat
CABOG	41	265	711	24	375	24	45	18
MINIMUS	205	31	89	44	382	37	208	36
PCAP	91	69	194	50	455	57	94	41
SUTTA	72	93	621	45	261	23	75	22
TIGR	69	111	357	31	1281	24	134	20

*Brucella suis* assemblies obtained with long reads have been compared using standard assembly statistics. We reported the assembler employed, the number of contigs returned by the assembler, the N50 length, the length of the longest contig and the number of mis-assemblies identified by *dnadiff*. Moreover we reported the number of features returned by *amosvalidate* and the number of such features that overlap with a real mis-assembly. The same data is reported for the ICA-features.

Last four columns of Table 2 show how, in general, by reducing the feature space we are able to discard a large number of features (in the TIGR case we pass from 1281 to 134 features) without discarding any significant number of valid features (*i.e.*, features that coincide with real mis-assemblies). This statistics on true discovery suggest that our method does not suffer from a lack of desirable sensitivity. It was noticed in [7] that assembly features have, in general, high sensitivity (higher than 98%) but they lack specificity. We also noticed that the situation remains true even after dimensionality reduction of the feature space. In general this is a consequence of how features arise in two scenarios: features that affect large portions of contigs and assembler-specific features. In the first scenario a feature affects a large portion of a contig when, however, only a relatively small fragment of such contig is a true mis-assembly. The second scenario is much more problematic, we noticed that some assemblers have a particular feature that appears almost in every contig (in the case of *Brucella suis*, LOW_GOOD_CVG appears in almost all TIGR contigs). When this feature is selected by the ICA analysis the specificity is deeply affected (however, the sensitivity remains high). This situation can be avoided by selecting the most representative features for each assembler, but a larger dataset of genomes is necessary in order to successfully apply PCA and ICA.

### Short Reads Results

As explained in the Materials and Methods Section, the real short read dataset is somewhat different from the simulated ones. In the real dataset, we used only four different genomes, sequenced with Illumina producing reads of different lengths. In order to obtain sufficiently many assemblies that could allow PCA and ICA to be performed, we extracted and assembled subsets of reads with different coverages. We selected four different kinds of reads, from which it is possible to obtain as general a set of PCs as possible. However it would have been preferable to obtain an even larger and more representative datasets that could have led to more accurate results. On the other hand, the simulated dataset was obtained by simulating genomes of 20 different organisms at a constant coverage (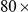), comprising paired reads of length 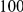 bp and insert size of 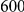 bp. The results obtained using this dataset gave us a picture of the state-of-the-art assembly capabilities. However, as seen in the analyses of long reads, PCs obtained through simulated data appear dissimilar to the real ones.

Again, PCA analysis on the real and simulated dataset (Table 3) suggested the presence of highly “over-dimensioned” feature space. While, to achieve just 80% of the variance, we need only 5 components in the real datasets, as few as 4 are adequate in the simulated ones. Using more sophisticated random matrix theory and the Marčenko-Pastur function, we observed that it is safe to disregard an extra PC with no loss of accuracy in either of the cases.

**Table 3:** More Informative Principal Components For Short Reads.

	Real			Simulated		
FEATURES	PC1	PC2	PC3	PC1	PC2	PC3
BREAKPOINT	-	-	-	-	-	-
COMPRESSION	−0.28	−0.15	0.24	0.32	0.20	0.33
STRETCH	−0.30	−0.11	0.32	0.2	0.37	0.26
HIGH_NORMAL_CVG	0.12	0.44	−0.09	0.15	0.13	−0.62
HIGH_OUTIE_CVG	−0.32	−0.33	−0.29	0.19	0.15	−0.536
HIGH_READ_COVERAGE	−0.26	−0.30	−0.41	0.35	0.09	−0.01
HIGH_SINGLEMATE_CVG	0.23	−0.26	−0.37	−0.11	−0.50	0.15
HIGH_SNP	−0.19	−0.05	−0.38	0.37	0.00	−0.06
HIGH_SPANNING_CVG	−0.07	−0.38	0.12	0.36	−0.24	−0.16
KMER_COV	−0.08	−0.22	0.47	0.31	0.28	0.28
LOW_GOOD_CVG	0.41	−0.32	0.09	0.34	−0.35	0.09
N50	−0.48	0.08	0.10	−0.19	0.25	0.02
NUM_CONTG	0.36	−0.41	0.12	0.30	−0.42	0.03
cumulative variation	26%	50%	63%	43%	62%	75%

First three PCs for the two short reads datasets: real short reads and, simulated short reads. At the bottom of each component we reported the cumulative variation represented.

As far as the first real PC is concerned, we saw how the LOW_GOOD_CVG and N50 are among the most important features. Again, as in the long reads datasets, the two features are negatively correlated. While in the first long read PC most of the features were positively correlated, in the short read case it was no longer true. We saw that compression and extension events (COMPRESSION, STRETCH) are correlated to mate-pairs problems (HIGH_OUTIE_CVG) while the number of contigs is positively correlated to areas with low coverage. These effects can be explained in the following way: areas with compression and extension events are likely to contain a large number of mis-oriented reads, while the production of an excess contigs can be a consequence of a failure in properly estimating the copy number of repeated sequences (thus resulting in a low coverage). The second PC distinguishes assemblies with high HIGH_NORMAL_CVG. All the other relevant features are negatively correlated to this one.

As expected, the PCs resulting from the simulated dataset differed to some degree from the ones obtained from real datasets. Also in this case N50 is negatively correlated and its coefficient is not among the maximal ones (like in the long read case). The first component is similar to the first component of the simulated long read dataset. In the second component the main source of variation between assemblies could be explained by a low number of contigs and regions covered only by unpaired reads as well as a large number of compression expansion events and mate pairs in different contigs.

Using ICA we extracted two feature subsets: one for the real data and the other for the simulated data. As before, we considered ICs that account for 80% of the kurtosis distribution. The ICA-space for the real dataset is formed by 6 features: COMPRESSION, LOW_GOOD_CVG, KMER_COV, HIGH_SPANNING_CVG, HIGH_OUTIE_CVG, and CE_STRETCH.

**Figure 4:**
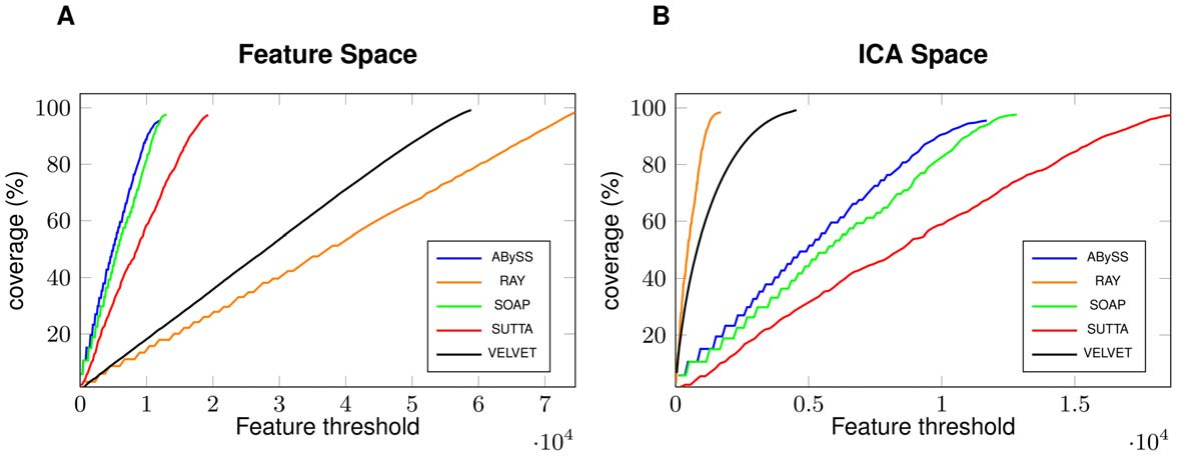
Feature Response Curve and ICA features: Real Short Reads. Figure A shows the FRC for the 5 assemblers on *E. coli* real dataset (read length 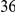 bp, insert size 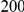 bp and coverage 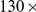) when using all the feature space. Figure B shows the FRC computed on the ICA-selected features.

In Figure 4 we draw the FRC for the *E. coli* dataset composed of paired reads of length 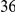 bp that form a 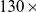 coverage of the sequenced genome. Figure 4A represents the FRC computed on all the feature space, while Figure 4B represents the FRC computed on the ICA-space. When all features are employed (Fig. 4A), we can clearly see how SUTTA, ABySS and SOAP outperform RAY and VELVET. This situation is in contrast with the analysis presented in Table 4, where we clearly see that RAY is the assembler generating very few mis-assemblies along with ABySS, SUTTA and SOAP all behaving similarly. VELVET has much larger number of mis-assemblies, suggesting that the long contigs that it produces are often a consequence of incorrect choices. If we reduce to the ICA-subspace (Fig. 4B) the picture changes drastically but some problems still remain: RAY, as one would expect, becomes the best assembler, but it is now surprisingly closely followed by VELVET. Moreover, ABySS becomes one of the worst assemblers. This situation is probably a consequence of the way in which features have been computed: ABySS and SOAP provide no facility but to map reads back to the contigs in order to build a layout (it is not clear if the other De-Bruijn based assemblers build a real Sanger-like layout or if they make use of heuristics). This indirect approach clearly skews our empirical analysis. Nonetheless, we can see how the reduced ICA space is able to highlight the good performances of RAY. The last four columns of Table 4 show that ICA-features significantly reduce the number of features to be considered (even if this time the reduction is not as impressive as the one obtained with long reads) without noticeably affecting the number of real features (with the only exception of VELVET). This picture again motivates us to highlight the need for assemblers to provide read layouts that could ensure a meaningful evaluation.

**Table 4:** Assembly Comparison Real Short Reads: E. coli 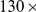.

Assembler	# Ctg	N50	Max (Kbp)	Errs	# Feat	# corr Feat	# ICA Feat	# corr ICA Feat
ABySS	113	97	268	11	11804	119	11475	105
RAY	194	58	140	17	74565	52	1701	30
SOAP	125	109	267	62	12254	174	12053	140
SUTTA	690	11	41	56	7949	140	5528	114
VELVET	65	142	428	136	2156	26	131	2

*E. coli* assemblies obtained with short real reads have been compared using standard assembly statistics. We reported the assembler employed, the number of contigs returned by the assembler, the N50 length, the length of the longest contig and the number of mis-assemblies identified by *dnadiff*. Moreover we reported the number of features returned by *amosvalidate* and the number of such features that overlap with a real mis-assembly. The same data is reported for the ICA-features.

**Figure 5:**
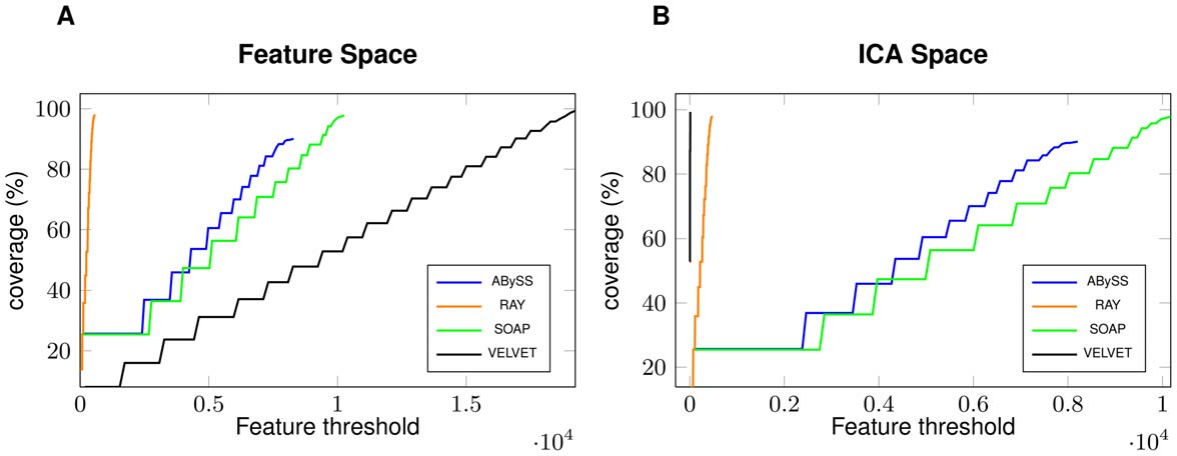
Feature Response Curve and ICA features: Simulated Short Reads. Figure A shows the FRC for 4 assemblers on *Brucella suis* simulated dataset (read length 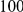 bp, insert size 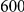 bp and coverage 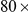) when using all the feature space. Figure B shows the FRC computed on the ICA-selected features.

In the short read case, we explored the ICA-features also for the simulated dataset too. We again selected 6 features: namely, HIGH_READ_COVERAGE, HIGH_SNP, HIGH_NORMAL_CVG, HIGH_SPANNING_CVG, KMER_COV, and STRETCH. Figure 5 demonstrates the differences between FRC curve computed on all the feature space (left part) and on the ICA-space (right part). As before, we observed a similar anomalous behaviour: VELVET produces the worst assembly, when evaluated with all the features taken into account, whereas it is the best assembler when only the ICA-features are considered (RAY, ABySS and SOAP do not show any significant variation). These pictures are in contrast with the data summarized in Table 5 where, again, we can see that RAY and ABySS are the assemblers less affected by mis-assemblies, while VELVET contains as many as 23 mis-assemblies. A closer scrutiny explained that VELVET has a large number of HIGH_SINGLEMATE_CVG (that are clear witnesses of a mis-assembled region) that are not taken into account in the ICA-space. This is a clear bias that affects the ICA analysis but it is difficult to estimate how much this depends on the read simulator or on the in-vitro generated layout.

**Table 5:** Assembly Comparison Simulated Short Reads: Brucella suis.

Assembler	# Ctg	N50	Max (Kbp)	Errs	# Feat	# corr Feat	# ICA Feat	# corr ICA Feat
ABySS	20	301	850	2	8250	67	8174	63
RAY	27	261	459	1	590	5	486	2
SOAP	30	299	843	15	10142	112	10057	108
VELVET	23	663	1010	22	13547	149	11	1

*Brucella suis* assemblies obtained with short simulated reads have been compared using standard assembly statistics. We reported the assembler employed, the number of contigs returned by the assembler, the N50 length, the length of the longest contig and the number of mis-assemblies identified by *dnadiff*. Moreover we reported the number of features returned by *amosvalidate* and the number of such features that overlap with a real mis-assembly. The same data is reported for the ICA-features.

### Conclusions

Classically sequence assembly problem has been formulated as a Shortest Common Superstring problem (SCSP), which has many undesirable implications: algorithm development in the shadows of intractability (NP-completeness), piecemeal quick fixes to the original imprecise formulation to address complex genome structures (*e.g.*, repeats), ad hoc measures to incorporate information from long-range data through scaffolding (*e.g.*, mate-pairs), and finally, forfeiture of precious haplotype information that has adversely affected subsequent GWAS. In this regime, now, there are a handful of heuristics (greedy, overlap-layout-consensus, and sequencing-by-hybridization), several dozen software pipelines, and hundreds of variations and versions that differ wildly in their preprocessing, parameters, and post-hoc analysis. Yet, accuracy and suitability of assemblies produced by any of these systems have remained largely unexamined. Attempts have been made to validate the results of an assembler either with simulated in silico data or with auxiliary information from genetic or physical maps, or by sequencing randomly selected short fragments. These approaches have not succeeded – either hampered by the inaccuracy of the tests themselves, or by the additional cost-overhead they could incur.

A different approach has now emerged from attempts to validate the assemblies by examining various features of read lay-outs, which could be indicative of sources of misassemblies. These features have also led to a new technique for assembly comparison using Feature-Response Curves (FRC). However, in the absence of a thorough and rigorous analysis of the structure of the feature-space, FRC analysis lacked a solid foundational grounding. This paper addresses these issues via principal and independent component analyses (PCA and ICA, respectively) that identify key features and how each of them is related to the other features. The paper cocluded that most features are redundant, few of the widely accepted features are not very good predictors of accuracy, and a small number of good features can be isolated relatively easily without affecting the methods sensitivity. These analyses have quantified our insight into why none of the existing assemblers has been satisfactory.

But further work needs to be done. Reducing the Feature-space through Independent Component Analysis does not solve the lack of specificity of the method. It is not clear if this is a consequence of the currently designed features and if new features can circumvent this problem. We identified as a major stumbling block in obtaining reliable results, the lack of NGS-based assemblers producing an assembly-layout. There are some fundamental (but unavoidable) biases in our techniques. For instance, how robust is our detected redundancy in the current set of features? A premature elimination of a feature, presumed redundant, could be imprudent, especially if it is later determined that the detected redundancy depended on the currently existing set of assembly algorithms that created the examples for the supervised learning. We have tried to avoid possible biases by selecting representative sets of algorithms from all three known paradigms: SBH (sequencing-by-hybridization), OLC (overlap-layout-consensus) and B&B (branch-and-bound). Currently, the only example from B&B (*e.g.*, exhaustive global optimization) is SUTTA (with some variations: aggressive vs. conservative), although, for the other classes, there are many more. In the near future, we expect to see more SUTTA-like algorithms (or even innovation of new assembly paradigms), which will require continual updating of the results reported here. In contrast, the idea of distilling an unbiased set of features of existing genomes (not algorithms) is likely to be less variable, since the samples of genomes used already contain a wide variety of structural elements. The set of features extracted in this way could lead to more realistic *in silico* genome models, which in turn can be used in training and assessing assembly algorithms – *e.g.*, identifying a set of features for existing genomes that expose classes of errors or inconsistencies, intrinsic to a specific assembler.

In the future, we expect to see more analysis of this nature, not just to understand the feature-space and its redundancy, but also to understand how newer algorithms, technologies (*e.g.*, next-next-generation sequencing, long-range mapping – including dilution, mate pairs, strobe reads, optical mapping, *etc.*), and testing strategies (based on simulation or better references) will affect not only the way old (negative) features are tamed and new features are invented, but also the redundancy structure of the feature-space itself. Related to these issues, there is also the question of how a subset of informative features can be learned, so that a global optimization formulation of the sequence assembly problem (in terms of few score and penalty functions involving these features) would lead to higher fidelity. Recently developed SUTTA assembly algorithm, based on branch-and-bound, is specifically designed to exploit such global formulations. There are also many thorny issues related to how to develop better in silico genome sequence simulation, benchmark datasets, data standards, and a trusted institution with the authority to validate genome assemblies.

## Supporting Information

Document S1 **Complete experiments description.** This document contains a detailed list of genomes used in the paper and the commands used to obtain the simulated data.
(PDF)

## References

[1] Narzisi G, Mishra B (2010). Scoring-and-Unfolding Trimmed Tree Assembler: Concepts, Constructs and Comparisons.. Bioinformatics (Oxford, England).

[2] Menges F, Narzisi G, Mishra B (2011). TOTALRECALLER : Improved Accuracy and Performance via Integrated Alignment & Base-Calling.. Bioinformatics (Oxford, England).

[3] Li R, Fan W, Tian G, Zhu H, He L (2009). The sequence and de novo assembly of the giant panda genome.. Nature.

[4] Nagarajan N, Pop M (2009). Parametric complexity of sequence assembly: Theory and applications to next generation sequencing.. Journal of Computational Biology.

[5] Lander ES, Linton LM, Birren B, Nusbaum C, Zody MC (2001). Initial sequencing and analysis of the human genome.. Nature.

[6] Lin Y, Li J, Shen H, Zhang L, Papasian C (2011). Comparative Studies of de novo Assembly Tools for Next-generation Sequencing Technologies.. Bioinformatics.

[7] Phillippy AM, Schatz MC, Pop M (2008). Genome assembly forensics: finding the elusive misassembly.. Genome biology.

[8] Narzisi G, Mishra B (2011). Comparing De Novo Genome Assembly: The Long and Short of It.. PLoS ONE.

[9] Miller J, Koren S, Sutton G (2010). Assembly algorithms for next-generation sequencing data.. Genomics.

[10] Earl DA, Bradnam K, St John J, Darling A, Lin D (2011). Assemblathon 1: A competitive assessment of de novo short read assembly methods.. Genome Research.

[11] Salzberg SL, Phillippy AM, Zimin AV, Puiu D, Magoc T (2011). Gage: A critical evaluation of genome assemblies and assembly algorithms.. Genome Research.

[12] Jolliffe I (2002). Principal Component Analysis, Second Edition.. Wiley Online Library.

[13] Hyvärinen A, Karhunen J, Erkki O (2001). Independent Component Analysis.. John Wiley & Sons, first edition.

[14] Lu H, Plataniotis K, Venetsanopoulos A (2011). A survey of multilinear subspace learning for tensor data.. Pattern Recognition.

[15] Imam I, Vafaie H (1994). An empirical comparison between global and greedy-like search for feature selection.. Proceedings of the Florida AI Research Symposium (FLAIRS-94), Pensacola Beach, FL.

[16] Bi J, Bennett K, Embrechts M, Breneman C, Song M (2003). Dimensionality reduction via sparse support vector machines.. The Journal of Machine Learning Research.

[17] Boutsidis C, Mahoney M, Drineas P (2008). Unsupervised feature selection for principal components analysis.. Proceeding of the 14th ACM SIGKDD international conference on Knowledge discovery and data mining.

[18] Prasad M, Sowmya A, Koch I (2004). Efficient feature selection based on independent component analysis.. Intelligent Sensors, Sensor Networks and Information Processing Conference, 2004. Proceedings of the 2004.

[19] Johnstone I (2006). High dimensional statistical inference and random matrices..

[20] Hyvärinen A, Oja E (1997). A fast fixed-point algorithm for independent component analysis.. Neural computation.

[21] Liu J, Pearlson G, Windemuth A, Ruano G, Perrone-Bizzozero N (2009). Combining fMRI and SNP data to investigate connections between brain function and genetics using parallel ICA.. Human brain mapping.

[22] Nahlawi LI, Mousavi P (2010). Single nucleotide polymorphism selection using independent component analysis.. Annual International Conference of the IEEE Engineering in Medicine and Biology Society IEEE Engineering in Medicine and Biology Society Conference.

[23] Miller JR, Delcher AL, Koren S, Venter E, Walenz BP (2008). Aggressive assembly of pyrosequencing reads with mates.. Bioinformatics (Oxford, England).

[24] Sommer DD, Delcher AL, Salzberg SL, Pop M (2007). Minimus: a fast, lightweight genome assembler.. BMC bioinformatics.

[25] Huang X, Yang SP (2005). Generating a genome assembly with PCAP.. Current protocols in bioinformatics/editoral board, Andreas D Baxevanis [et al] Chapter.

[26] Sutton G, White O, Adams M, Kerlavage A (1995). TIGR Assembler: A new tool for assembling large shotgun sequencing projects.. Genome Science and Technology.

[27] Richter DC, Ott F, Auch AF, Schmid R, Huson DH (2008). MetaSim: a sequencing simulator for genomics and metagenomics.. PloS one.

[28] Zerbino D, Birney E (2008). Velvet: algorithms for de novo short read assembly using de Bruijn graphs.. Genome research.

[29] Boisvert S, Laviolette F, Corbeil J (2010). Ray: Simultaneous Assembly of Reads from a Mix of High-Throughput Sequencing Technologies.. Journal of Computational Biology.

[30] Simpson J, Wong K, Jackman S, Schein J (2009). ABySS: A parallel assembler for short read sequence data.. Genome.

[31] Li R, Zhu H, Ruan J, Qian W, Fang X (2010). De novo assembly of human genomes with massively parallel short read sequencing.. Genome research.

