# Supplementary material for: Feature-by-Feature – Evaluating De Novo Sequence Assembly
Source: PLoS One. 2012 Feb 3;7(2):e31002. doi: 10.1371/journal.pone.0031002 (PMC3272011; doi:10.1371/journal.pone.0031002)
Supplement: Document S1 — Complete experiments description. This document contains a detailed list of genomes used in the paper and the commands used to obtain the simulated data. (PDF) [file pone.0031002.s001.pdf]

Feature Analysis to Evaluate *De novo* Sequence Assembly (Supplemental Material)

Francesco Vezzi, Giuseppe Narzisi, Bud Mishra

August 5, 2011

1 Datasets

1.1 Real Long Read Dataset

Description of the real long read dataset. All the genome projects have been downloaded from `ftp://ftp.ncbi.nih.gov/pub/TraceDB/`. For each project we downloaded the fasta files, the quality files and the ancillary data (read trimming, paired reads constraints, etc.).

Sample	Genome	Length	# reads	Avg length	tot length	coverage
1	Alcanivorax	3789834	39044	1080	42177431	11.13
2	Alteromonas macleodii	4448980	43878	1007	44209050	9.94
3	Bacillus anthracis	5227293	125879	854	107563457	20.58
4	Bacillus cereus	5269030	68503	1071	73375574	13.93
5	Bifidobacterium dentium	2636367	28240	757	21394408	8.12
6	Bordetella bronchiseptica	5339179	55895	946	52909812	9.91
7	Bbradyrhizobium	8264687	89675	1018	91346484	11.05
8	Brucella Suis	3315173	36275	895	32499069	9.8
9	Burkholderia mallei	5742303	101634	1008	102506338	17.85
10	Candidatus korarchaeum	1590757	30168	1048	31625328	19.88
11	Escherichia coli	5572075	58534	1119	65538509	11.76
12	Lactobacillus gasseri	2011295	42477	882	37495317	18.64
13	Mesoplasma florum	793224	86566	788	68278119	86.08
14	Shewanella oneidensis	4969803	69499	752	52307472	10.53
15	Staphylococcus apidermidis	2616530	57997	900	52208201	19.95
16	Staphylococcus aureus	2809421	50035	818	40937267	14.57
17	Thioalkalivibrio	3464554	28458	940	26766873	7.73
18	Vibrio cholerae b33	4154698	30570	1075	32865241	7.91
19	West nile virus	11029	3148	937	2952302	267.69
20	Wolbachia sp	1267782	26816	981	26332465	20.77
21	Yersinia pestis biovar	4681648	73065	989	72291428	15.44

The datasets have been assembled using CABOG, MINIMUS, PCAP, SUTTA, TIGR that require different input formats. In order to produce the input files for the assemblers we implemented the following pipeline:

- with `tarchive2ca` we converted the downloaded project into CABOG input format (*frg* file);
- with `toAmos` we converted the *frg* into an *afg* that can be handled by SUTTA and MINIMUS:
- with `frg2ta` we converted the *frg* into TIGR input format;
- with a script we converted the TIGR format into a PCAP like input format;

1.2 Real Short Read Datasets

The four real short read datasets have been downloaded from the Short Read Archive website.

	Name	SRA	Length	# reads	Avg length	coverage	ins length
1	Escherichia coli	SRX000429	4639675	20816447	36	161.52	200
2	Chlamydia trachomatis	ERX012723	1042579	8100845	54	419.58	243
3	Staphylococcus aureus ST239	ERX012594	2906507	5307429	75.73	138.29	268
4	Yersinia pestis KIM	SRX048908	4600755	2311795	100	50.25	300

In order to produce a number of assemblies able to perform the statistical analysis we assembled the following different coverages:

- `Escherichia coli`: 30×,50×,70×,90×,110×,130× and, 160×;
- `Chlamydia trachomatis`: 30×,50×,70×,90×,110×,130×;
- `Staphylococcus aureus`: 30×,50×,70×,90×,110×,130×;
- `Yersinia  pestis`: 30× and 50×;

We assembled E.coli and Chlamydia datasets with ABySS, Ray, SUTTA, SOAP and Velvet, while Staphylococcus and Yersinia datasets have been assembled using only ABySS, Ray, SOAP and Velvet.

1.3 Simulated Datasets

The reference sequences used to generate the simulated datasets have been downloaded from `http://www.ncbi.nlm.nih.gov/genome`

			Long Reads		Short Reads	
	Name	Length	read length	coverage	read length	coverage
1	Alcanivorax borkumensis	3120143	800	12	100	80
2	Alteromonas macleodii	4448980	800	12	100	80
3	Bacillus amyloliquefaciens	3918589	800	12	100	80
4	Bacillus cereus	5269030	800	12	100	80
5	Bordetella bronchiseptica	5339179	800	12	100	80
6	Brucella suis	3315173	800	12	100	80
7	Burkholderia mallei NCTC	5742303	800	12	100	80
8	Campylobacter jejuni	1777831	800	12	100	80
9	Chlamydia trachomatis	1038842	800	12	100	80
10	Chlorobium tepidum	2154946	800	12	100	80
11	Dehalococcoides	1413462	800	12	100	80
12	Geobacter metallireducens	3997420	800	12	100	80
13	Mesoplasma florum	793224	800	12	100	80
14	Shewanella oneidensis	5131416	800	12	100	80
15	Staphylococcus aureus COL	2813862	800	12	100	80
16	Staphylococcus aureus JH1	2906507	800	12	100	80
17	Staphylococcus epidermidis	2643840	800	12	100	80
18	Thioalkalivibrio sulfidophilus	3464554	800	12	100	80
19	Wolbachia	1267782	800	12	100	80
20	Yersinia pestis	4600755	800	12	100	80

In order to simulate long reads we used MetaSim software. In particular we used the following command line:

```
MetaSim cmd -r NUM_READS -f INS -t VAR --sanger
--sanger-mean LENGTH --sanger-param2 0 REFERENCE
```

where `NUM_READS` was set to the number of reads required to achieve the desired coverage. For each sequence we produce a 10× coverage composed by reads of length 800 with insert size of 3000 bp and a standard variation of 500 bp, and a 2× coverage composed by reads of length 800 with insert size of 10000 bp and a standard variation of 2000 bp.

For what concerns the short read simulations we used SimSeq, the read generator used for Assemblathon 1 (www.assemblathon.org), to produce an 80× coverage formed by paired reads of length 100 bp and insert size of 600 bp:

```
java -jar -Xmx2048m  SimSeq.jar -1 100 -2 100 --error ERR1 --error2 ERR2
     --insert_size 600 --insert_stdev 100 --read_number PAIRS
       --reference REFERENCE -o OUTPUT  --read_prefix read
```

where ERR1 and ERR2 are the precomputed errors models for the first and the second read.
